# Prevalence and associated risk factors of dry eye disease in Hotan, Xinjiang: a cross-sectional study

**DOI:** 10.1186/s12886-023-02955-9

**Published:** 2023-05-15

**Authors:** Xiaolong Li, Zhen Wang, Jingyu Mu, Hamila Puerkaiti, Ayinu Nulahou, Jie Zhang, Yong Zhao, Qi Sun, Yuanyuan Li, Yan Wang, Yunxian Gao

**Affiliations:** 1 Xinjiang Uygur Autonomous Region Traditional Chinese Medicine Hospital, No.116, Yellow River Road, Shayibake District, Ürümqi, 830000 China; 2 School of Public Health, Xinjiang Medical University, 393 Xinyi Road, Xinshi District, Ürümqi, Xinjiang China; 3 Department of Ophthalmology, Traditional Chinese Medicine Hospital of Xinjiang Uyghur Autonomous Region, 116 Huanghe Road, Shayibake District, Ürümqi, Xinjiang China

**Keywords:** Dry eye disease, Prevalence, Risk factors, Hotan

## Abstract

**Objectives:**

To assess the prevalence of dry eye disease (DED) in the Uyghur population in Hotan, Xinjiang, and to identify risk factors associated with this disorder.

**Methods:**

Between January and September of 2020, 5,121 Uyghur subjects aged 18 − 98 years from 105 villages were selected and studied cross-sectionally using a whole-group random sampling method in the Hotan area, Xinjiang, China. The Ocular Surface Disease Index questionnaire was used to collect subjective symptoms of DED and examine tear-film break-up times. The break up time and Schirmer’s test were used to collect objective signs, to determine the prevalence of DED and its risk factors.

**Results:**

A total of 5,121 subjects aged 18 − 98 years were recruited from the Uyghur population in the Hotan region of Xinjiang, China, for eye examinations and questionnaire surveys. A total of 40.6% (2,078/5,121) were diagnosed with DED, of which 38.3% were male and 41.9% were female. The prevalence of DED was the highest in subjects ≥ 65 years of age, with 47.8% in males and 53.3% in females. The lowest occurrence was in subjects 18 − 44 years of age, with 32.5% in males and 33.7% in females. Older age, tea drinking, and staying awake late were risk factors affecting the severity of DED prevalence (*p* < 0.05), but there was no significant difference in sex, presence of diabetes, or presence of hypertension (*p* > 0.05).

**Conclusion:**

The prevalence of DED in the study population was 40.6%, and its prevalence was higher in females, when compared with males. The prevalence of dry eye also increased with age, and at an advanced age, female sex, smoking, staying awake late, and not exercising were risk factors for DED.

## Introduction

The TFOS Dry Eye Workshop II (TFOS DEWS II) defines dry eye disease (DED) as a chronic ocular surface disease caused by multiple factors, involving an unstable tear film or imbalance in the ocular surface microenvironment due to abnormalities in the quality, quantity, and kinetics of tears, which can be accompanied by ocular surface inflammatory responses, tissue damage, and neurological abnormalities, resulting in multiple ocular discomfort and/or visual dysfunction [1]. The prevalence of DED ranges from 5 to 50% according to global surveys [2–7], with a higher prevalence in Asia than in Europe and the United States. The variability in the prevalence of DED is mainly influenced by geographic location, population, and different diagnostic criteria. Numerous epidemiological surveys have shown that female sex, older age [8], and dry and extreme climates are risk factors for the development of dry eye [9].

Xinjiang is located in northwestern China, in the interior of the Asian and European continents, and has a distinct temperate continental climate due to its distance from the ocean, and being surrounded by high mountains that prevent easy access to oceanic air currents. In 2017, Yingying [10] reported the prevalence of DED in the Uyghur population in Kashgar (21.8%), but the sample size of this study was relatively small. Related studies have shown that DED is significantly associated with environment and ethnicity [11], but there has been no large sampling of the DED population in the Uyghur population, which is the predominant population in the Hotan region in the south of Xinjiang. This region is close to the Taklamakan Desert, which has an arid climate. In the present study, we therefore conducted a survey of DED, lifestyles, and diets in the Uyghur population in the Hotan region of Xinjiang, to determine the prevalence and risk factors for DED. Based on our findings, we propose health measures to reduce the occurrence of DED in this population.

## Subjects and methods

### Subjects

Uyghur subjects aged ≥ 18 years of age in the Hotan area of Xinjiang were selected from January to September of 2020 using whole-group random sampling in villages. Inclusion criteria included: (1)ages ≥ 18 years; (2) Duration of residence > 1 year; (3)voluntary participation in the study; (4)signing an informed consent form. Exclusion criteria included: (1)those with skin diseases, such as Stevens-Johnson syndrome and Aspergillosis; (2)subjects wearing contact lenses;(3)subjects previously treated with ophthalmic surgery, such as refractive surgery, glaucoma, or cataract surgery;(4)subjects with active ocular surface diseases; and (5) patients who could not cooperate due to impaired consciousness, psychological disorders, or psychiatric disorders.

### Methods

(1)Questionnaire The Ocular Surface Disease Index (OSDI), which was translated into Uyghur, was used to analyze the distribution of main symptoms based upon the frequency of reported ocular discomforts. Each point was calculated according to the duration of the symptom, with 4 points for all the time, 3 points for most of the time, 2 points for half of the time, 1 point for a small part of the time, and 0 points for never occurring. The final OSDI score was calculated as (total score of questions answered × 100)/(number of questions answered × 4). The total OSDI score was 100, with a higher score suggesting a more serious ocular surface disease and less stable tear film. This scoring system has been shown to be efficient, objective, and accurate. The Schirmer’s test is a non-anesthetic test that reflects the secretory function of the main lacrimal gland (physiological secretion). Fluorescein staining tear film breakup time (FBUT) is the most commonly used method in clinical practice. It must be performed at room temperature, under suitable humidity, and in a light-proof room. The standard test method involves applying 1% sodium fluorescein solution (2 μL) to the conjunctival sac with a sterile dropper, or to touch the lid margin of the lower eyelid with fluorescein paper moistened with antibiotic drops but without excess residual solution. The subject then blinks 3 ~ 4 times so that fluorescein is applied to the surface of the eye, with both eyes looking straight ahead. The results obtained in ≤ 5 s indicate whether the degree of dry eye is moderate to severe.

### Diagnostic criteria

The study complied with the dry eye diagnostic criteria in the Chinese Dry Eye Expert Consensus: Examination and Diagnosis (2020) [12]:1. Patients complained of subjective symptoms such as ocular dryness, foreign body sensation, burning sensation, fatigue, discomfort, eye redness, or fluctuating visual acuity, with a score of ≥ 7 using the Chinese Dry Eye Questionnaire or the Ocular Surface Disease Index (OSDI index ≥ 13). Subjects with a FBUT ≤ 5 s or NIBUT (noninvasive tear break-up time) < 10 s or Schirmer’s test (without anesthesia) ≤ 5 mm/5 min were diagnosed with dry eye. Because there is no international classification of dry eye severity, this study graded dry eyes as mild, moderate, and severe according to OSDI questionnaire scores, with OSDI scores of 13 − 22, 23 − 32, and 33 − 100, respectively [7]. For all tests of dry eye used in this study, both eyes were examined and data from both eyes were recorded. When there was a difference between two eyes, data from the worse eye was selected for statistical analysis.

### Statistical analysis

Excel 2016 software was used to establish the database, and SPSS statistical software for Windows, version 26.0 (SPSS, Chicago, IL, USA) was used for statistical analysis. Data are expressed as percentages (%), and confidence intervals were calculated for the prevalence of dry eye and risk factors. Comparisons between groups were made using the chi-square test or Fisher’s exact test according to the conditions, and the associations between DED and its influencing factors were analyzed using the one-way chi-square test and multi-factor logistic regression. A value of *p* < 0.05 was considered a statistically significant difference.

## Results

### Basic characteristics of the subjects

A total of 5,121 Uyghur adult subjects were selected from the Hotan area, Xinjiang, China, with an average age of 51.7 ± 10.3 years, with the largest number of subjects in the 45 − 54 years age range, with 1,722 (33.6%) subjects in this age group. There were 1,905 (37.2%) males and 3,216 (62.8%) females; 2,462 (48.1%) drank tea, and 913 (17.8%) had late hours. A total of 913 (17.8%) had the habit of staying up late, 213 (4.2%) had diabetes mellitus, and 1,278 (25.0%) had hypertension. Table 1 shows specific information on the basic characteristics of the subjects.

**Table 1:** Basic characteristics of the subjects (*N* = 5,121)

Variable	No.(%)of respondents
Age (years)	
18 ~	1458(28.5%)
45 ~	1722(33.6%)
55 ~	1250(24.4%)
65 ~	691(13.5%)
Gender	
Male	1905(37.2%)
Female	3216(62.8%)
Life style	
Smoking habits	492(9.6%)
Tea drinking habits	2462(48.1%)
Drinking habits	421(8.2%)
Habit of staying up late (sleeping after 24:00)	913(17.8%)
Exercise habits	259(5.1%)
Disease	
Diabetes	213(4.2%)
Hypertension	1278(25.0%)

### Prevalence of DED in different age groups and sexes

Among 5,121 subjects, 3,515 (68.6%) had an OSDI questionnaire score ≥ 13, 2,368 (46.2%) had a BUT score ≤ 5, and 1,057 (20.6%) had a Schirmer’s test score ≤ 5, with 2,078 cases of dry eye with a prevalence of 40.6%. Subjective symptoms (OSDI ≥ 13), objective sign1 (BUT ≤ 5), objective sign2 (Schirmer’s test ≤ 5), and prevalence of DED significantly differed among age groups (all, *p* < 0.05). With increasing age subjective symptoms (OSDI ≥ 13), objective sign1 (BUT ≤ 5), and objective sign2 (Schirmer’s test ≤ 5) of DED showed a trend toward greater severity and a higher prevalence of DED. By stratifying by sex, it was found that the subjective symptoms of DED (OSDI ≥ 13), objective signs of dry eye1 (BUT ≤ 5), objective signs of dry eye2 (Schirmer’s test ≤ 5), and the prevalence of DED differed among age groups in males and females (all *p* < 0.05), and the overall trend was consistent with dry eye symptoms becoming more severe with increasing age, as shown in Table 2.

**Table 2:** The prevalence (%) of dry eye disease and symptoms by age and sex

	N	OSDI ≥ 13 N(%)	BUT ≤ 5 N(%)	Schirmer ≤ 5 N(%)	DED N(%)
Age groups					
18 ~	1458	933(64.0%)	546(37.4%)	243(16.7%)	486(33.3%)
45 ~	1722	1209(70.2%)	787(45.7%)	348(20.2%)	690(40.1%)
55 ~	1250	873(69.8%)	642(51.4%)	301(24.1%)	554(44.3%)
65 ~	691	500(72.4%)	393(56.9%)	165(23.9%)	348(50.4%)
Total	5121	3515(68.6%)	2368(46.2%)	1057(20.6%)	2078(40.6%)
χ^2^/p		21.880/ < 0.001*	90.147/ < 0.001*	27.702/ < 0.001*	66.612/ < 0.001*
Male					
18 ~	461	294(63.8%)	167(36.2%)	69(15.0%)	150(32.5%)
45 ~	579	403(69.6%)	237(40.9%)	102(17.6%)	206(35.6%)
55 ~	495	350(70.7%)	235(47.5%)	108(21.8%)	196(39.6%)
65 ~	370	269(72.7%)	196(53.0%)	89(24.1%)	177(47.8%)
Total	1905	1316(69.1%)	835(43.8%)	368(19.3%)	729(38.3%)
χ^2^/p		9.037/0.029*	28.036/ < 0.001*	13.984/0.003*	22.893/ < 0.001*
Female					
18 ~	997	639(64.1%)	379(38.0%)	174(17.5%)	336(33.7%)
45 ~	1143	806(70.5%)	550(48.1%)	146(21.5%)	484(42.3%)
55 ~	755	523(69.3%)	407(53.9%)	193(25.6%)	358(47.4%)
65 ~	321	231(72.0%)	197(61.4%)	76(23.7%)	171(53.3%)
Total	3216	2199(68.4%)	1533(47.7%)	689(21.4%)	1349(41.9%)
χ^2^/p		13.072/0.004*	73.286/ < 0.001*	17.999/ < 0.001*	54.094/ < 0.001*

^*^Represents a *p* value of less than 0.05, which is considered statistically significant

**Fig. 1:**
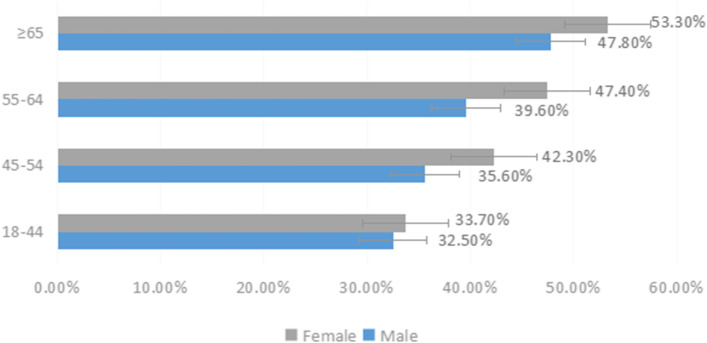
Prevalence by sex in each age group

**Fig. 2:**
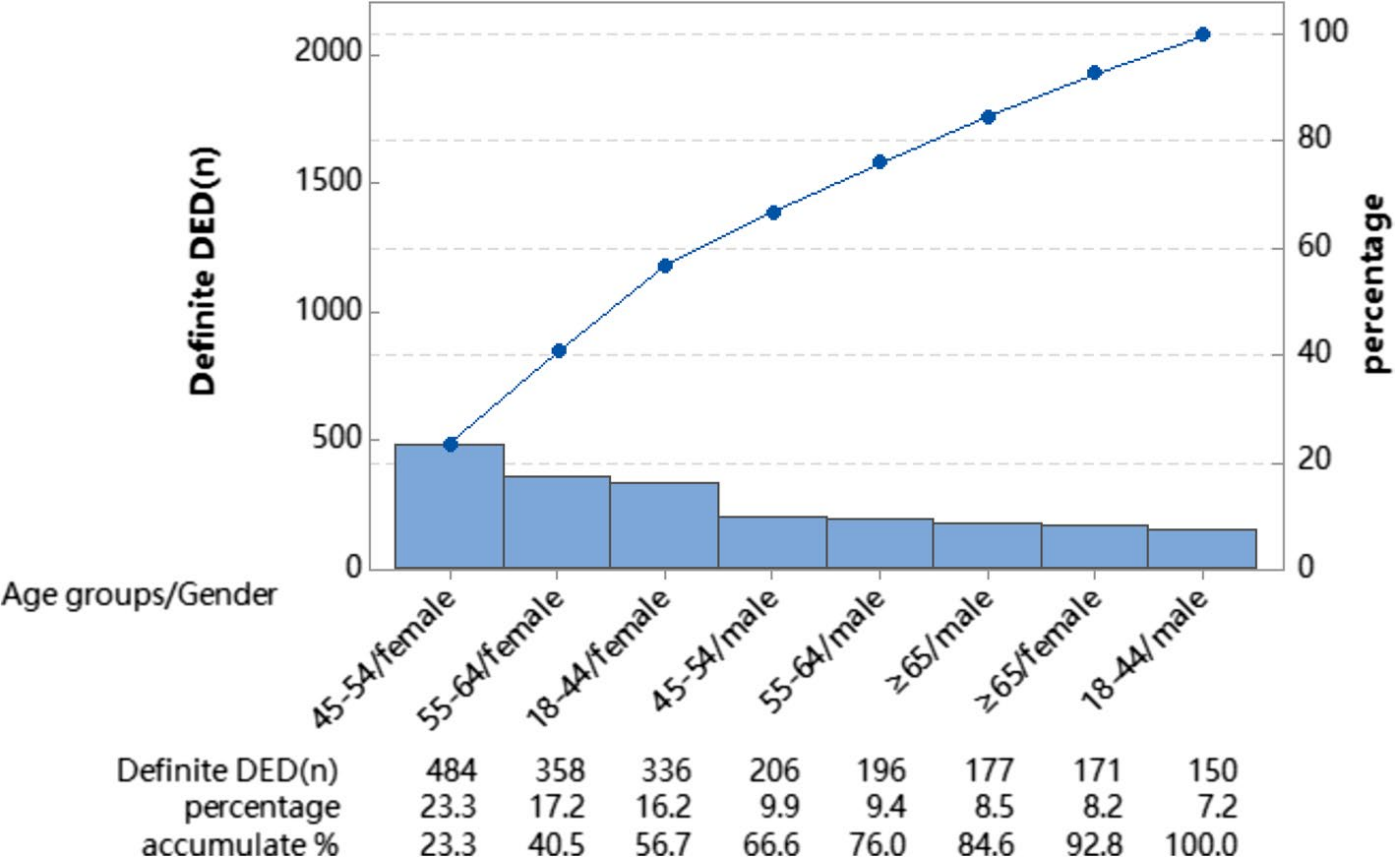
Number of patients in different age groups and sexes

The prevalence of DED in males and females was highest in the age group ≥ 65 years, with occurrence of 47.8% and 53.3%, respectively; in the age group of 18 − 44 years, the prevalence of dry eye was lowest in both males and females, with 32.5% and 33.7%, respectively; in all age groups, the prevalence of dry eye in males was lower than that in females, as shown in Fig. 1. Pareto diagrams were constructed, showing that the main affected groups were 45 − 54 years of age (females), 55 − 64 years of age (females), 18 − 44 years of age (females), 45 − 54 years of age (females), and 55 − 64 years of age (males) in that order, with a cumulative compositional ratio of 76%, with middle-aged subjects and females as the main groups (Fig. 2).

### Univariate analysis of DED

Among all subjects, the prevalence of DED was 38.3% in males and 41.9% in females, with the prevalence of DED lower in males than in females (*p* < 0.05). In addition, the prevalence of DED was higher in the Uyghur population with those drinking tea, with late hours, and without regular exercise (all *p* < 0.05); but there was no significant difference between the prevalence of DED in the diabetic population and the non-diabetic population (*p* > 0.05), and the prevalence of DED in the population with hypertension was significantly higher than that in the population without hypertension (*p* < 0.05) (Table 3).

**Table 3:** Demographic characteristics by dry eye disease status

	N	Diagnosed-DED N(%)	Non-DED N(%)	χ2/ Fisher’s exact probability method	*P* Value
Sex				6.715	0.010*
Male	1905	729(38.3%)	1176(61.7%)		
Female	3216	1349(41.9%)	1867(58.1%)		
Life style					
Smoke	492	209(42.5%)	283(57.5%)	0.816	0.366
Not smoke	4629	1869(40.4%)	2760(59.6%)		
Tea	2462	1041(42.3%)	1421(57.7%)	5.714	0.017*
Don’t drink tea	2659	1037(39.0%)	1622(61.0%)		
Drinking	421	160(38.0%)	261(62.0%)	1.260	0.262
No drinking	4700	1918(40.8%)	2782(59.2%)		
Stay up late	913	437(47.9%)	476(52.1%)	24.463	< 0.001*
Don’t stay up late	4208	1641(39.0%)	2567(61.0%)		
Exercise	259	83(32.0%)	176(68.0%)	8.235	0.004*
Don’t exercise	4862	1995(41.0%)	2867(59.0%)		
Disease					
Diabetes	213	92(43.2%)	121(56.8%)	0.630	0.427
Without diabetes	4908	1986(40.5%)	2922(59.5%)		
Hypertension	1278	566(44.3%)	712(55.7%)	9.721	0.002*
Without hypertension	3843	1512(39.3%)	2331(60.7%)		
Total	5152	2078(40.6%)	3043(59.4%)		

^*^Represents a *p* value of less than 0.05, which is considered statistically significant

### DED according to OSDI classification

**Fig. 3:**
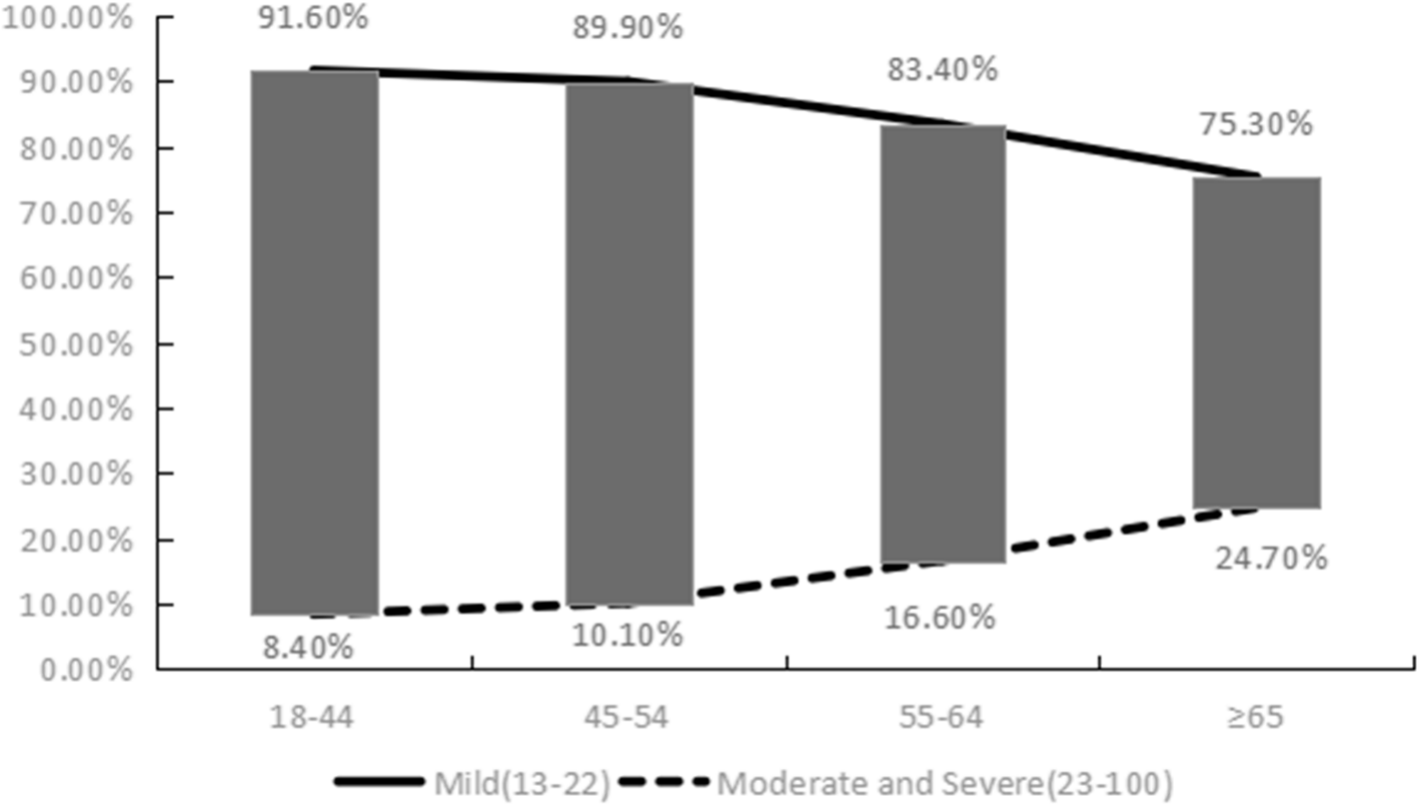
Distribution of mild, moderate, and severe dry eye in different age groups

The 2,078 Uyghur subjects with DED were divided into mild dry eye, moderate dry eye, and severe dry eye according to OSDI scores, among which 1,789 subjects (86.1%) had mild dry eye, 267 (12.8%) moderate dry eye, and 22 subjects (1.1%) had severe dry eye. The severity of DED differed among age groups, whether they drank tea, and whether they had late hours (all *p* < 0.05), but there was no significant difference among sexes, whether they had diabetes, and whether they had hypertension (*p* > 0.05), as shown in Table 4. The highest percentage of mild dry eye (91.6%) and the lowest percentage of mild dry eye (75.3%) were found in the age group ≥ 65 years of age, while the percentage of moderate and severe dry eye showed a significant increasing trend with age (Fig. 3).

**Table 4:** Dry eye grading according to the Ocular Surface Disease Index

	Mild dry eye n (%)	Moderate dry eye n (%)	Severe dry eye n (%)	χ^2^/ Fisher’s exact probability method	*P* Value
	(OSDI:13–22)	(OSDI:23–32)	(OSDI:33–100)		
Age				60.860	< 0.001*
18 ~	445(91.6%)	36(7.4%)	5(1.0%)		
45 ~	620(89.9%)	63(9.1%)	7(1.0%)		
55 ~	462(83.4%)	86(15.5%)	6(1.1%)		
65 ~	262(75.3%)	82(23.6%)	4(1.1%)		
Sex				3.675	0.159
Male	624(85.6%)	101(13.9%)	4(0.5%)		
Female	1165(86.4%)	166(12.3%)	18(1.3%)		
Life style					
Smoke	181(86.6%)	25(12.0%)	3(1.4%)	0.461	0.794
Not smoke	1608(86.0%)	242(12.9%)	19(1.0%)		
Tea	888(85.3%)	133(12.8%)	20(1.9%)	14.818	0.001*
Don’t drink tea	901(86.9%)	134(12.9%)	2(0.2%)		
Drinking	138(86.3%)	20(12.5%)	2(1.3%)	0.077	0.962
No drinking	1651(86.1%)	247(12.9%)	20(1.0%)		
Stay up late	367(84.0%)	59(13.5%)	11(2.5%)	11.588	0.003*
Don’t stay up late	1422(86.7%)	208(12.7%)	11(0.7%)		
Exercise	73(88.0%)	9(10.8%)	1(1.2%)	0.499	0.694
Don’t exercise	1716(86.0%)	258(12.9%)	21(1.1%)		
Disease					
Diabetes	81(88.0%)	10(10.9%)	1(1.1%)	0.418	0.767
Without diabetes	1708(86.0%)	257(12.9%)	21(1.1%)		
Hypertension	472(83.4%)	85(15.0%)	9(1.6%)	5.581	0.061
Without hypertension	1317(87.1%)	182(12.0%)	13(0.9%)		
Total	1789(86.1%)	267(12.8%)	22(1.1%)		

^*^Represents a value of *p* < 0.05, which is considered statistically significant

### Multifactorial analysis of DED

**Fig. 4:**
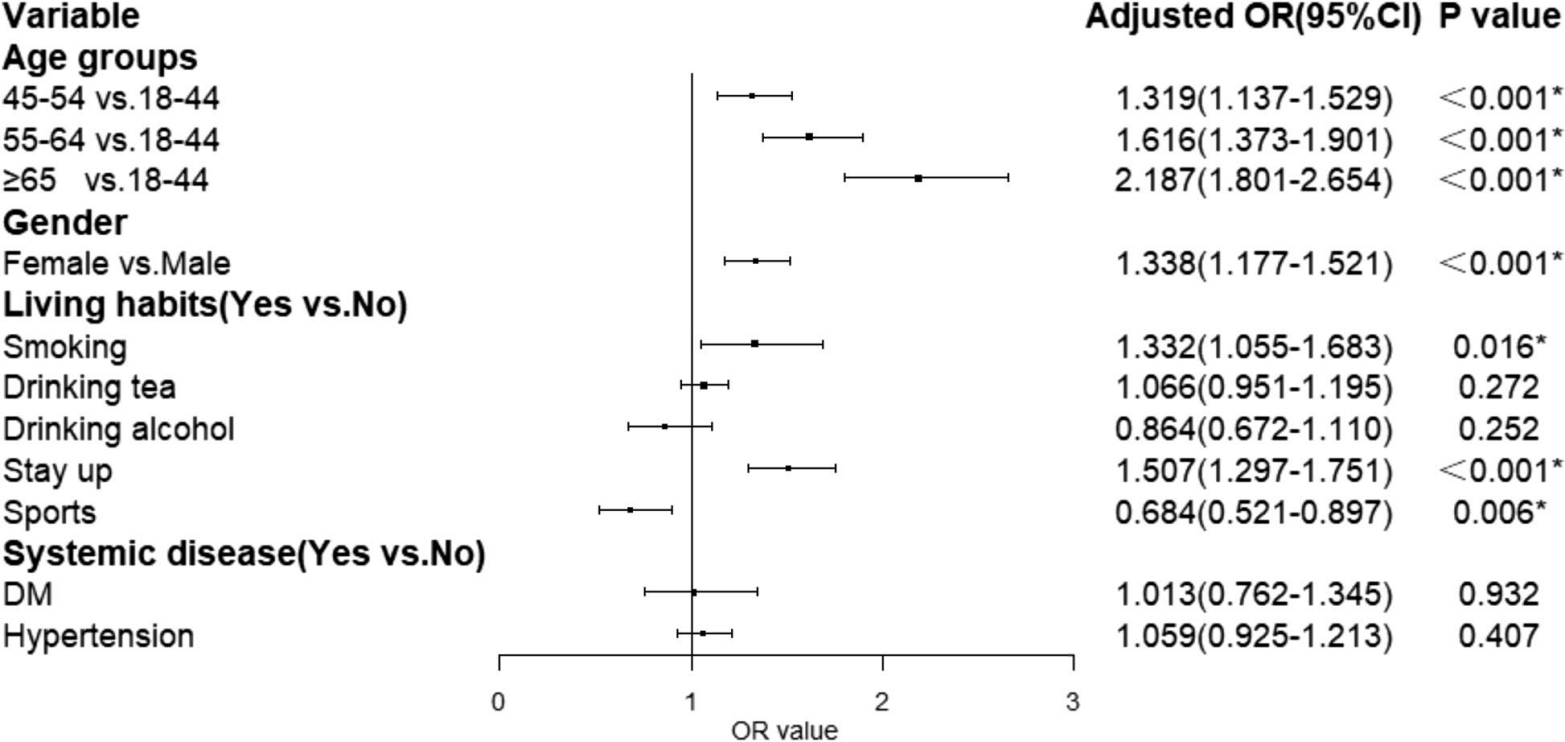
Logistic regression analysis of risk factors associated with definite dry eye disease. ^*^Represents a value of *p* < 0.05, which is considered statistically significant

A multifactorial logistic regression analysis of DED showed that age, female [odds ratio (OR): 1.338;95%; confidence interval (CI): 1.177 − 1.521], smoking (OR: 1.332; 95% CI: 1.055 − 1.683) and late hours (OR: 1.507; 95% CI: 1.297 − 1.751) were risk factors for DED. Exercise (OR: 0.684; 95% CI: 0.521 − 0.897) was a protective factor for DED. The prevalence of DED increased with age, with those 45 − 54 years of age, 55 − 64 years of age, and those ≥ 65 years of age being 1.3 times (95% CI: 1.137 − 1.529), 1.6 times (95% CI: 1.373 − 1.901), and 2.2 times (95% CI: 1.801 − 2.654) more likely to develop dry eye than those 18 − 44 years of age, respectively. DED was not significantly associated with drinking tea, alcohol consumption, diabetes, or hypertension (all *p* > 0.05), as shown in Fig. 4.

## Discussion

In this study, we found that the prevalence of DED in the Uyghur population in rural areas of Hotan, Xinjiang was 40.6% (2078/5121), which was higher than the prevalence of dry eye in Kashgar, Xinjiang, which was 27.8% [10], and significantly higher than the overall prevalence of dry eye in China, which was 17% [13], but slightly lower than the prevalence of dry eye in Africa (42.0%) [14], and significantly higher than in countries such as Europe and the United States [15–17]. The large variation in reported DED prevalence across the globe may be related to differences in diagnostic criteria, parameters used, study methods, and population ethnicities. Previous studies have reported that climate [18] and high latitude are important risk factors for DED [11], and the Hotan region, located in the southern corner of the Xinjiang Uyghur Autonomous Region, is a typical inland arid zone, located in the interior of the Eurasian continent, with a warm arid desert climate, strong sunshine, scarce precipitation throughout the year, windy and sandy weather, and strong ultraviolet light, which are all environmental factors that can lead to DED [18, 19].

In the present study, we showed, using symptom data, that the overall prevalence of DED was 40.6%, while the prevalence of symptomatic DED (i.e., OSDI ≥ 13) was 68.4%, a prevalence similar to 69% in Palestine [7], but higher than those reported in Jordan and the Arab region [6, 20], which may be related to similar latitudes, arid climates, and proximities to the desert. In our study, we found increasing trends in the prevalence of subjective symptoms and signs of DED with increasing age. The prevalence of DED was higher in females than males in this study (41.9% vs. 38.3%), with the highest prevalence in males and females ≥ 65 years of age, with 47.8% and 53.3%, respectively. These findings confirmed that in females, increasing age is a risk factor for the development of DED, which may be associated with decreasing estrogen levels [8].

The 2,078 Uyghur patients with DED were classified according to OSDI scores into mild dry eye, moderate dry eye, and severe dry eye, with 1,789 cases (86.1%) with mild dry eye, 267 cases (12.8%) with moderate dry eye, and 22 cases (1.1%) with severe dry eye. The variabilities in the severities of DED were associated with factors such as age, drinking tea, and late hours, with no significant difference in the distributions of sex, diabetes, or hypertension. The percentages of patients with mild DED decreased gradually with age, with the highest percentage of mild DED (91.6%) in those 18 − 44 years of age and the lowest percentage of mild DED (75.3%) in those ≥ 65 years, while the percentages of patients with moderate and DED showed a significant increasing trend with age. Our results indicated that subjects who smoked, had late hours, and did not exercise tended to have higher percentages of DED. One study reported a significant effect of green tea consumption on ocular tear film quality [21], yet another study reported that green tea extract was an effective, safe, and well-tolerated topical treatment for mild and moderate evaporative DED [22], so the effect of tea consumption on DED needs further study. Ayaki concluded that approximately half of patients with DED had poor sleep habits, and symptoms of DED, especially pain, were also associated with sleep quality [23], and insomnia impaired the function of the lacrimal system and induced DED [24]. A Japanese study also found that lack of physical activity, prolonged sedentary behavior, and use of video terminals were associated with increased susceptibility to DED in middle-aged and elderly Japanese subjects [25].

This is associated with the local consumption of greasy foods and less vegetables, and dyslipidemia is a high risk factor for DED. The prevalence of metabolic syndrome is high in Xinjiang, especially among ethnic minorities because of their special dietary patterns [26, 27]. Metabolic syndrome is associated with a systemic pro-inflammatory state and leads to high tear osmolarity, which activates inflammatory pathways and initiates cytokine release, leading to corneal damage and loss of cupped cells [28], which is associated with a local diet high in fat and low in vegetable consumption, and dyslipidemia was a high risk factor for DED [29–31]. Other studies of the Mediterranean diet showed that olive oil and nuts [32], omega-3 and omega-6 unsaturated fatty acids [33, 34], and honey-related products [35, 36] were beneficial for patients with DED, and this diet and natural foods (broccoli, saffron, nuts, and walnuts) may have potential antioxidant, anti-inflammatory, and neuroprotective effects [37], but the mechanisms need further study. The Mediterranean diet has also been shown to be beneficial in systemic metabolic diseases and cardiovascular disorders [38]. It has also been confirmed that aerobic exercise promotes tear production and improves tear film stability in patients with DED [39]. Determining whether the prevention and control of diseases can be achieved through rational improvement of dietary patterns and healthy lifestyles is therefore significant and deserves long-term studies.

Finally, our study was based on a Uyghur population with a large sample size and included objective examinations and questionnaires. However, the study may have some limitations. The use of Schirmer’s test and FBUT examination were invasive, which may have affected the true state of tears and tear film, although a subsequent study included a dry eye analyzer to provide more objective data analysis and analysis of the Meibomian gland [40].

In conclusion, the prevalence of DED was high among Uyghurs in the Hotan region of Xinjiang, and was especially common among elderly patients and females. Subjects in this region were not aware of the negative impact of DED on the visual quality and quality of life. With the increasing trends of aging and popularity of video terminals(VDT), the prevalence of DED in this region may increase. We therefore need to increase social awareness about DED to reduce modifiable risk factors (arid environment, high fat diet, and systemic diseases such as diabetes [1, 26, 27]. However, we also need to investigate other potential risk factors associated with DED to better prevent and control the occurrence of this disorder.

## Data Availability

All data supporting the study is presented in the manuscript or available upon request from the corresponding author of this manuscript.

## Abbreviations

CI: Confidence intervals
DED: Dry Eye Disease
DEWS 2017: International Dry Eye Workshop
FL/S: Fluorescein cornealstaining
OSDI: Ocular Surface Disease Index
SPSS: The statistical Package for Social Sciences
TBUT: Tear filmbreak-up time
VDT: Video display terminals
NIBUT: Noninvasive tear break-up time
FBUT: Film breakup time
